# A robust energy management system for Korean green islands project

**DOI:** 10.1038/s41598-022-25096-3

**Published:** 2022-12-20

**Authors:** Lilia Tightiz, Joon Yoo

**Affiliations:** School of Computing, Gachon University, 1342 Seongnamdaero, Seongnam-si, Gyeonggi-do 13120 Korea

**Keywords:** Electrical and electronic engineering, Engineering, Energy grids and networks, Power distribution

## Abstract

Penetration enhancement of renewable energy sources is a core component of Korean green-island microgrid projects. This approach calls for a robust energy management system to control the stochastic behavior of renewable energy sources. Therefore, in this paper, we put forward a novel reinforcement learning-driven optimization solution for the convex problem arrangement of the Gasa island microgrid energy management as one of the prominent pilots of the Korean green islands project. We manage the convergence speed of the alternating direction method of multipliers solution for this convex problem by accurately estimating the penalty parameter with the soft actor-critic technique. However, in this arrangement, the soft actor-critic faces sparse reward hindrance, which we address here with the normalizing flow policy. Furthermore, we study the effect of demand response implementation in the Gasa island microgrid to reduce the diesel generator dependency of the microgrid and provide benefits, such as peak-shaving and gas emission reduction.

## Introduction

To combat global warming, substituting RESs for fossil fuels is more beneficial to the island regions of the earth. As global warming continues, oceans rise, and islands disappear^1^. On the other hand, energy storage systems (ESS) mitigate the random nature of RESs, allowing microgrid power networks to take over the island’s power supply without relying on central power plants^2,3^.

In Korea, with 471 residential islands, using a microgrid to electrify the islands is the most cost-effective solution. In this country, 171 residential islands electrify with stand-alone microgrids, whereas DGs supplied their power earlier^4^. According to the Korean government plan, 63.8 GW of RESs will be installed by 2030. This amount is 20% of national electricity generation and would result in a 37% reduction in $CO_{2}$ emissions^5^. Implementing the Green Islands project is a significant step in this direction. One of the seven pilot green islands is Gasa Island, which initially operated as a stand-alone microgrid powered by three DGs^6^.

Due to the objective of the Korean green island project, Gasa Island has moved towards entirely relying on RESs with assistance from ESSs. In this self-sufficient microgrid, DGs were used only as backup power in case of contingency. However, it is impossible to achieve this goal without an intelligent, robust EMS unit that can coordinate energy supply resources performance to reduce greenhouse gases. Since the use of ESSs on this island, in addition to the compensation of RESs power absence, is the voltage and frequency regulation, large-scale ESSs are needed. A RES’s primary contribution on this island is to reduce greenhouse gas emissions, not to reduce residents’ electricity bills^4^. Meanwhile, introducing DR to lower the peak-average ratio reduces the need for further investment in ESSs and RESs besides minimizing DG utilization in emergencies. As a result, in this paper, we examine the energy management of Gasa Island as part of the Green Island project, which aims to implement economic dispatch as a baseline, and then develop EMS using DR programs to reduce DG usage and ultimately greenhouse gas emissions. According to these objectives and the constraints of high penetration of RESs in green islands, in addition to the uncertainty in loads, the Gasa island EMS problem is stochastic, high-dimensional, and sequential. The MG EMS arrangement should consider several factors based on the type of MG, including centralized or decentralized decision making, optimization methods, RESs output, and load uncertainty management. So far, MG EMS optimization has been carried out using a mathematical approach, meta-heuristics, and artificial intelligence (AI)^7^. These EMS scheduling may follow a centralized or decentralized manner. A real-time EMS for microgrid in a decentralized fashion is presented in^8^. This paper examined the MG voltage and frequency stability from a power electronics perspective. Economic model predictive control (EPMC) offered economic dispatch for residential microgrids in^9^. Authors in this paper addressed applying prediction error in RESs output in their model with stochastic MPC in the future attempt. However, it is costly and complex to implement MPC. However, uncertainty in RESs power production is crucial to be considered to provide an accurate EMS vastly in literature implemented by deploying probability distribution. Raghav et al.^10^ hired a sparrow search algorithm to arrange EMS, considering forecasting errors of RESs output and load prediction. They compared their method with a wide range of other metaheuristic-based optimization methods. Uncertainty in PV and WT power prediction is considered in the optimization of microgrid EMS^11^ by deploying different probability density functions. The authors of this paper used the quantum teaching learning-based (QTLBO) optimization method and compared the results with a real-coded genetic algorithm, differential evolution, and TLBO. This paper focuses on the robust arrangement of fast converged optimization methods to support online EMS. Another goal is to reduce DG roles to strengthen the $\hbox {CO}_{2}$-free island target through DR. Additionally, this paper deploys historical data of RESs output power and load in the modeling environment considering their randomness behavior.

ADMM is a widely used response to EMS arrangement as a complex problem since of deploying distributed computational approach to reduce the severity of high-dimensional characteristics of microgrid EMS problem^12^. ADMM solved the quadratic and non-quadratic format of the economic dispatch problem of the islanded microgrid in^13^. The proposed algorithm showed high performance in balancing power consumption and generation of the island microgrid. Lyu et al. in^14^ proposed a dual-consensus ADMM to provide less communication-dependent microgrid economic dispatch scheduling. ADMM was used to solve the microgrid economic dispatch considering ESS costs in^15^ and^16^. However, a primary concern of hiring ADMM is the hyper-parameter dependency of its convergence to the optimum. Designing dynamic step-size for updating dual variables and converting ADMM to proximal gradient method are remedies has been offered to overcome the convergence hindrance of ADMM^17–20^.

Recently, considerable literature has grown up around the theme of sequential prediction of deep learning^21^ and in an augmented horizon deep reinforcement learning (DRL) to estimate the penalty parameter of ADMM^22,23^. DRL proved the fast convergence provision of ADMM by justifying penalty parameters in^22^. However, the utilized DRL method was TD3 which suffers from hyper-parameter dependency and worsens the large-scale vulnerability of solving convex problems such as microgrids EMS. Zeng et al.^23^ solved the quadratic format of distributed optimal power flow with the help of Q-learning to estimate the penalty parameter of ADMM. By transferring the residual value variant to the reward function in each iteration, the authors in this paper solved the sparse reward problem of arranged reinforcement learning. Moreover, this paper hired reinforcement learning, i.e., Q-learning’s discrete action space limited the more credited actions searching possibilities. Therefore, in this paper, we employ the state-of-the-art of SAC to offer a fast and accurate converged ADMM. In contrast to other methods that experience the local optimum trap after a certain number of iterations due to ineffective exploration, SAC supports continuous action space and adds stochasticity to the policy, providing excellent action exploration that persists until the last training process iterations^24^. We deploy the NFP technique to increase the density of the policy probability distribution to overcome the sparse reward issue. The contribution of this paper has four folds:

- Providing a convex problem arrangement of Gasa island EMS considering DR and load flow constraints to make profits for consumers and the utility grid.
- Arrangement of a SAC-based solution to estimate the penalty parameter of ADMM to support the high-dimension and complex problem of Gasa island EMS.
- Solving sparse reward hindrance with a less computational burden on the learning process of SAC algorithm by arranging high-density action space with the NFP approach.
- Exploration of less dependency on conventional generators and acquisition costs with DR implementation.

The remainder of this paper is organized as follows. By specifying the objective function and microgrid elements constraints, “Problem formulation” section formulates the problem. “Proposed method” section represents the solution method, and “Results and discussion” section investigates the novelty of the proposed solution by analysis of the results and compare with benchmark methods. Later on, “Conclusion” section discloses the most relevant conclusions of our work.

## Problem formulation

### Microgrid objective function

**Figure 1:**
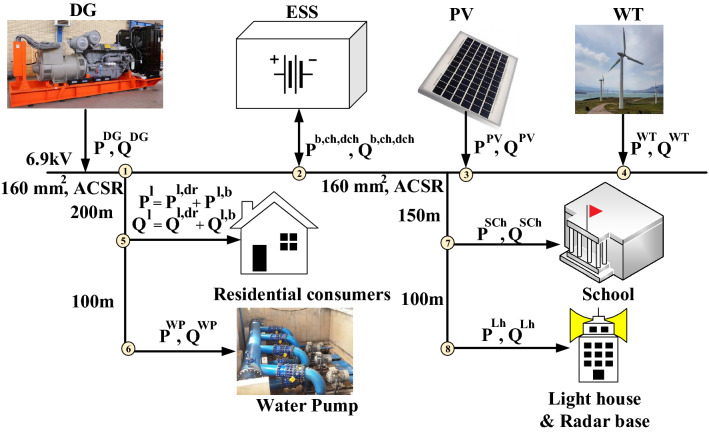
Gasa island microgrid structure.

In this paper, we consider two scenarios for the EMS arrangement of the Gasa island microgrid. The first scenario includes photovoltaic cells (PV), wind turbines (WT), DGs, ESSs, and loads, as shown in Fig. 1. In the second scenario, we schedule the DR for the residential load to decrease the peak-average ratio. This approach reduces DG consumption and carbon emission production, making the green island a more practical objective. Consequently, we define the objective function of the Gasa island microgrid as minimizing power generation costs for the first scenario, and we enhance that by attaching minimization of power consumption cost for consumers through DR implementation. We formulate the objective function of the Gasa island microgrid, considering two scenarios as follows.

1 $$\begin{aligned} min \Bigg (\sum _{t=1} ^T \sum _{i=1} ^N (c_i^g P_{i,t}^g + c_i^s u_t^s)+\sum _{t=1} ^T \sum _{j=1} ^M P_{t,j}^L price_{t,j} \Bigg ), \end{aligned}$$

where i: The number of generation units; j: The number of loads; T: The time period of optimization; $c^g$: The cost of power generation (KRW); $P^g$: The amount of power generation (kW); $c^s$: The cost of start-up and shut-down of conventional power generation units (KRW); $u^s$: Conventional power generation units on/off status; $P^L$: The amount of power consumption (kW); *price*: The price of load power consumption (KRW).

The first term of (1) calculates the cost of power generation, and the second part is the power consumption expenses. According to Fig. 1, $C^g$ can be applied to power generation units that include PV, WT, DG, and ESS in discharging mode. The cost of start-up and shut-down applies to conventional units, which is DG in this study. Since we plan to reduce peak load through implementing DR, consumption cost minimization is meaningful for this program participant loads.

### Microgrid elements modeling and constraints

The main objective of $\mathrm{CO}_2$ reduction in green island advent encourages priority of RESs in supplying loads. Therefore, we decline the cost of RESs power generation. The only RESs constraint is the maximum amount of power that can produce.

2 $$\begin{aligned}&0 \le P^{PV}(t) \le P^{PV}_{max}, \end{aligned}$$

3 $$\begin{aligned}&0 \le Q^{PV}(t) \le Q^{PV}_{max}, \end{aligned}$$

4 $$\begin{aligned}&0 \le P^{WT}(t) \le P^{WT}_{max}, \end{aligned}$$

5 $$\begin{aligned}&0 \le Q^{WT}(t) \le Q^{WT}_{max}, \end{aligned}$$

where $P^{PV}_{max}$ and $Q^{PV}_{max}$ are the maximum active/reactive power generation of PVs. $P^{WT}_{max}$ and $Q^{WT}_{max}$ are WTs’ highest amount of active/reactive power production.

DG is the only conventional generator in the Gasa island microgrid. In addition to its limitations concerning the amount of energy generated, the DG faces constraints regarding its working duration and variation in power output as follows.

6 $$\begin{aligned}&P^{DG}_{min} \le P_{DG}\le P^{DG}_{max}, \end{aligned}$$

7 $$\begin{aligned}& \left( {P^{{DG}} (t)} \right)^{2}  + \left( {Q^{{DG}} (t)} \right)^{2}  \le \left( {S_{{max}}^{{DG}} } \right)^{2} (t),  \end{aligned}$$

8 $$\begin{aligned}& \left( {T_{{on}}^{{DG}} (t) - T_{{up}}^{{DG}} } \right)\left( {u^{{DG}} (t) - u^{{DG}} (t + 1)} \right) \ge 0,  \end{aligned}$$

9 $$\begin{aligned}& \left( {T_{{off}}^{{DG}} (t) - T_{{down}}^{{DG}} } \right)\left( {u^{{DG}} (t + 1) - u^{{DG}} (t)} \right) \ge 0,  \end{aligned}$$

where $T^{DG}_{up}$ and $T^{DG}_{down}$ determine minimum up and down time, respectively. $u^{DG}$ is a binary value that shows the DG is on or off. The DG’s power generation cost is calculated according to (10).

10 $$\begin{aligned} c^{DG}(t) = a_1 + a_2 P^{DG} + a_3 {\left(P^{DG}\right)}^2, \end{aligned}$$

where $a_1$, $a_2$, and $a_3$ are factors for the fuel cost of DG. We also consider start-up cost of DG ($c^{s,DG}$) as a fixed number of 10 KRW.

The followings represent the main constraints that attach to the ESSs performance.

11 $$\begin{aligned}&SoC(t) = SoC(t-1)+\left(P^{b,ch}{\eta }^{ch}-\frac{P^{b,dch}}{{\eta }^{dch}}\right)\tau , \end{aligned}$$

12 $$\begin{aligned}&SoC_{min} \le SoC(t) \le SoC_{max}, \end{aligned}$$

13 $$\begin{aligned}&-P^{b,dch}_{min} \le P^{b}(t) \le P^{b,ch}_{max}, \end{aligned}$$

where ${\eta }^{ch}$ and ${\eta }^{dch}$ are ESS charging and discharging efficiency. $SoC_{min}$ and $SoC_{max}$ determine up and down limits of state of charge (SoC), and $\tau$ is the time slot. This study estimates the battery degradation cost based on (14).

14 $$\begin{aligned}&c^{b}(t)=\sigma \tau \big ( P^b(t)+E^b(t)\eta ^{leakage} \big ), \end{aligned}$$

15 $$\begin{aligned}&\sigma =c^{b,inv}/(E^{b,rated}n^{ch,dch}), \end{aligned}$$

where $\hbox {E}^{\mathrm{b,rated}}$ and $\hbox {E}^{\mathrm{b(t)}}$ are rated stored energy and available energy of battery at time t, respectively. $\eta ^{\mathrm{leakage}}$ determines leakage loss, $\sigma$ is a coefficient to calculate battery degradation during its lifespan, $\hbox {c}^{\mathrm{b,inv}}$ is the initial investment to provide battery, and $\hbox {n}^{\mathrm{ch,dch}}$ is the number of full charge and discharge cycles.

Gasa island includes 164 households^25^. To model the residential load, we hire the Enertalk open dataset that consists of the per appliance load consumption of 22 houses in Korea^26^. This dataset provides commonly deployed appliances’ power consumption data of a Korean household, including refrigerator, Kimchi refrigerator, water purifier, rice cooker, washing machine, and TV. We aggregated the power consumption of appliances by around 13% based on^27^ to estimate the heating and cooling system. We implemented DR on residential consumers to decrease the peak-average ratio. Therefore, we categorize the Enertalk appliance records into non-controllable, shiftable, and reducible loads. TV, Kimchi refrigerator, refrigerator, and rice cooker are non-controllable loads. The washing machine and heating and cooling system are shiftable and reducible loads, respectively. Therefore the electricity consumption of the heating and cooling system during the DR program based on inside building temperature ($Temp^{in}$) has the following restrictions^28,29^.

16 $$ \begin{aligned}&{\Delta }Temp^{in}(\tau )= \frac{1}{\beta \gamma }\left(Temp^{out}(t-1)-\beta P^{H \& C}(t)\right), \end{aligned}$$

17 $$\begin{aligned}&Temp^{in}_{min}\le Temp^{in}(t) \le Temp^{in}_{max}, \end{aligned}$$

18 $$   \begin{aligned}&P^{H \& C}_{min}\le P^{H \& C}(t) \le P^{H \& C}_{max}, \end{aligned}$$

where $\Delta$ $Temp^{in}$($\tau$) is the inside building temperature deviation during time step $\tau$. $Temp^{in}_{min}$ and $Temp^{in}_{max}$ are the inside building maximum and minimum desirable temperature. $\beta$ and $\gamma$ are the building thermal capacitance (kWh/ $^{\circ }$C) and reactance ( $^{\circ }$C/kW), respectively. $P^{H \& C}$(t) is the power usage of the heating or cooling system in time t.

To calculate the cost of load, we consider the time of use (TOU) price ($p_{t}^{TOU}$) according to Table 1^30^.

**Table 1:** TOU price ($p_{t}^{TOU}$) (KRW/kWh) based on KEPCO regulations.

TOU plan	Spring and Autumn		Summer		Winter	
	Time of day	Average price (KRW/kWh)	Time of day	Average price (KRW/kWh)	Time of day	Average price (KRW/kWh)
On-peak	10–12 a.m. and 13–17 p.m.	91.9	10–12 a.m. and 13–17 p.m.	157.7	10–12 a.m. and 17–20 p.m. and 22–23 p.m.	137.3
Off-peak	0–9 a.m. and 23–24 p.m	55.4	0–9 a.m. and 23–24 p.m	55.4	0–9 a.m. and 23–24 p.m	62.3
Mid-peak	Others	70.2	Others	101	Others	98

To provide a trade-off between utility profit in decreasing peak-average ratio with higher TOU in peak hours and consumer comfort, we add the anxiety rate term (${\mathcal {A}}_{rate}$) to the cost function to respect consumers’ preferred power consumption rate.

In the case of the heating and cooling system, the DR affects the customer comfort where the inside temperature ($Temp^{in}$(t)) exceeds limitations that are defined in (17)^31^.

19 $$ \begin{aligned} {\mathcal {A}}_{rate}^{H \& C}~~ = {\left\{ \begin{array}{ll} 0, &{}\quad Temp^{in}_{min}\le Temp^{in}(t) \le Temp^{in}_{max},\\ K e^{K (Temp^{in}-Temp^{in}_{max})},&{}\quad Temp^{in}> Temp^{in}_{max},\\ K e^{K (Temp^{in}-Temp^{in}_{min})},&{}\quad Temp^{in}< Temp^{in}_{min}, \end{array}\right. } \end{aligned}$$

where K is the anxiety rate coefficient.

Additionally, according to the customers’ preferred time of using the washing machine, the anxiety rate of the washing machine (${\mathcal {A}}_{rate}^{ WM}$) participating in DR is determined as follows.

20 $$\begin{aligned} {\mathcal {A}}_{rate}^{WM} = {\left\{ \begin{array}{ll}0, &{}\quad h_{s}<h_{WM}<h_{f},\\ \sigma ^{h_{s}-\zeta },&{}\quad h_{WM}<h_{s}, h_{WM}>h_{f}, \end{array}\right. } \end{aligned}$$

where $h_{s}$ and $h_{f}$ are the lower and upper preferred times of using the washing machine, respectively. $\sigma$ and $\zeta$ determine penalty coefficients of shifting washing machines out of consumers’ preferred time.

We deploy the water station load profile from^32^ and simulate school daily power consumption from^33^. The amount of lighthouse and radar base power consumption of Gasa island in the average daily load profile decline in this paper. During power dispatch scheduling, we consider load flow constraints for each branch of the Gasa island microgrid as follows.

21 $$\begin{aligned}& P_{i}^{g} (t) - P_{i}^{l} (t) = \sum\limits_{{i = 1}}^{n} {V_{i} } (t)V_{j} (t)\left( {G_{{i,j}} Cos\theta _{{i,j}}  + B_{{i,j}} Sin\theta _{{i,j}} } \right),  \end{aligned}$$

22 $$\begin{aligned}&{Q^{g}_{i}(t)}-{Q^{l}_{i}(t)}=\sum _{i=1}^{n}V_iV_j \left(G_{i,j}Sin\theta _{i,j}-B_{i,j}Cos\theta _{i,j}\right), \end{aligned}$$

where,

23 $$\begin{aligned}&{P^{g}_{i}(t)} = P^{DG}_{i} (t) + P^{PV}_{i} (t) + P^{WT}_{i} (t) + P^{b,ch}_{i} (t) - P^{b,dch}_{i} (t), \end{aligned}$$

24 $$\begin{aligned}&{P^{l}_{i}(t)} = P_{i}^{SCh}(t)+P_{i}^{WP}(t)+P_{i}^{Lh}(t)+P_{i}^{l,b}+P_{i}^{l,dr}, \end{aligned}$$

25 $$  \begin{aligned}&{P^{l,dr}_{i}(t)} = P_{i}^{H \& C}(t) + P_{i}^{WM}(t)- \Delta P_{i}^{H \& C,dr}(t) - \Delta P_{i}^{WM,dr}(t), \end{aligned}$$

26 $$\begin{aligned}&{Q^{g}_{i}(t)} = Q_{i}^{DG}(t)+ Q^{PV}_{i} (t) + Q^{WT}_{i} (t) + Q^{b,ch}_{i} (t) - Q^{b,dch}_{i} (t), \end{aligned}$$

27 $$\begin{aligned}&{Q^{l}_{i}(t)} = Q_{i}^{SCh}(t)+Q_{i}^{WP}(t)+Q_{i}^{Lh}(t)+Q_{i}^{l,b}+Q_{i}^{l,dr}, \end{aligned}$$

28 $$  \begin{aligned}&{Q^{l,dr}_{i}(t)} = Q_{i}^{H \& C}(t) + Q_{i}^{WM}(t)- \Delta Q_{i}^{H \& C,dr}(t) - \Delta Q_{i}^{WM,dr}(t), \end{aligned}$$

29 $$\begin{aligned}&V^{i}_{min}\le V^{i}(t) \le V^{i}_{max}, \end{aligned}$$

where i and j are bus indexes. $P^{i,j}(t)$ and $Q^{i,j}(t)$ are active and reactive power flow of lines between i and j buses at time t. $V^{i}_{min}$ and $V^{i}_{max}$ are lower and upper voltage limitations of each bus. $P^{SCh}$, $P^{WP}$, and $P^{Lh}$ are the school, water pump station, and lighthouse active power consumption, respectively. $P^{l,b}$ denotes non-DR participants’ residential load, and $P^{l,dr}$ is DR participants’ residential load. $\Delta P^{H \& C,dr}$, and $\Delta P^{WM,dr}$ are the amount of heating and cooling systems and washing machines’ active power consumption that contributes to DR. We modify the objective function in (1) based on Gasa island microgrid constraints as follows.

30 $$   \begin{aligned}{} & Min \left (\sum _{t=1} ^T \left(c^{DG} P_{t}^{DG} + c^b P_{t}^b + c^{s,DG} u_t^{DG}\right)+ \sum _{t=1} ^T P_{t}^L p_{t}^{TOU} \right )\nonumber \\ & =  Min \left(\sum _{t=1} ^T \left(a_1 + a_2 P^{DG} + a_3 {(P^{DG})}^2 + \alpha ~{(SoC(t)-SoC(t-1))}^2 P_{t}^b +c^{s,DG} u_t^{DG}\right) \right. \\ & \quad \left. + \sum _{t=1} ^T \left(P_{t}^{SCh}+P_{t}^{WP}+P_{t}^{Lh}+P_{t}^{H \& C} + P_{t}^{WM}- \Delta P_{t}^{H \& C}{\mathcal {A}}_{rate}^{H \& C,dr} -\, \Delta P_{t}^{WM}{\mathcal {A}}_{rate}^{WM,dr}\right) p_{t}^{TOU} \right ), \end{aligned}$$

subject to (2)–(9), (12), (13), (16)–(29). This formulation arranges a quadratic form of convex problem, which is solved in the following section based on ADMM technique.

## Proposed method

### ADMM

ADMM is a popular and reliable approach for convex quadratic programming. The conventional ADMM method attempts to solve the following problem^34^:

31 $$\begin{aligned} Min f(x) + g(x^{\prime }), ~~~~~~~~~~x\in {\mathbb {R}}^{n},x^{\prime }\in {\mathbb {R}}^{n^{\prime }}, \end{aligned}$$

subject to

32 $$\begin{aligned} Ax + A^{\prime }x^{\prime } = d,~~~~~~~~A\in {\mathbb {R}}^{p\times {n}}, A^{\prime }\in {\mathbb {R}}^{p\times n^{\prime }}, d \in {\mathbb {R}}^{p}, \end{aligned}$$

where x and $x^{\prime }$ are the optimization variables, f and g are coefficient matrices, d is the sequence, and f(x) and g($x^{\prime }$) are convex functions of optimization variables.

We arrange the Lagrangian function for the convex problem (31) as follows.

33 $$\begin{aligned} L(x, x^{\prime }, \lambda ) = f(x) + g(x^{\prime }) + {\lambda }^{T}(Ax + A^{\prime } x^{\prime } - d)+ \frac{\rho }{2}{\parallel }Ax + A^{\prime }x^{\prime }-d{\parallel }^2,~~\lambda \in {\mathbb {R}}^{n^{\prime \prime }}, \end{aligned}$$

where $\lambda$ is a relevant vector of multiplier for constraints (32), and $\rho$ is the penalty factor.

ADMM iteratively updates optimization variables according to the following.

34 $$\begin{aligned}&x_{k+1} = \underset{x}{argmin}~L_{\rho }(x,x^{\prime }_k, \lambda _k), \end{aligned}$$

35 $$\begin{aligned}&x^{\prime }_{k+1} = \underset{x^{\prime }}{argmin}~L_{\rho }(x_k,x^{\prime }, \lambda _k), \end{aligned}$$

36 $$\begin{aligned}&\lambda ^{k+1} = \lambda _{k} + \rho (Ax_{k+1} + A^\prime x^{\prime }_{k+1}-d), \end{aligned}$$

This iterative procedure will converge when the primal and dual residuals of ADMM techniques meet their thresholds $\epsilon _p$ and $\epsilon _d$, respectively, as follows.

37 $$\begin{aligned}&\parallel r_k^p\parallel _2 \le \epsilon _p , \end{aligned}$$

38 $$\begin{aligned}&\parallel r_k^d\parallel _2 \le \epsilon _d , \end{aligned}$$

where $\parallel r_k^p\parallel$ and $\parallel r_k^p\parallel$ represent the primal and dual residuals of the ADMM technique, calculated according to (39) and (40), respectively.

39 $$\begin{aligned}&\parallel r_k^p\parallel = Ax_{k} + A^{\prime }x^{\prime }_k - d, \end{aligned}$$

40 $$\begin{aligned}&\parallel r_k^d\parallel = 2 \rho A^T A^{\prime }(x^{\prime }_k - x^{\prime }_{k-1}). \end{aligned}$$

We deploy the decompose technique introduced in^23^ to solve our ADMM problem. To this end, we consider two penalty parameter vectors as follows.

41 $$\begin{aligned}&\rho _{PQ} \in \{\rho ^{PQ}_1, \rho ^{PQ}_2,\ldots , \rho ^{PQ}_{n_{PQ}}\}, \end{aligned}$$

42 $$\begin{aligned}&\rho _{V\theta } \in \{\rho ^{ V\theta }_1, \rho ^{ V\theta }_2, \ldots , \rho ^{V\theta }_{n_{V\theta }} \}, \end{aligned}$$

where $\rho _{PQ}$ is the penalty parameter for active and reactive power and $\rho _{V \theta }$ denotes voltage and phase angle penalty parameter. $n_{PQ}$ and $n_{V\theta }$ are the number of constraints for each pair of active and reactive power and voltage and phase angle, respectively.

Since penalty parameters are determinant factors in ADMM dual and primal in each iteration to converge according to (37) and (38), the sequential characteristic of these parameters calculation encourages using the DRL method to estimate penalty parameters.

### SAC algorithm with enhanced exploration

SAC is an actor-critic DRL method with sample efficiency specification of its off-policy approach^24^. SAC works in both discrete and continuous environments. The main characteristic of SAC is stochastic-based policy optimization by adding entropy to policy. This characteristic gives advantages of productive exploration and, consequently, a higher convergence rate to off-policy methods, such as deep deterministic policy gradient (DDPG) and twin delayed DDPG (TD3). Its sample efficiency due to learning from experience saved in the replay buffer is superior to on-policy techniques such as proximal policy optimization (PPO) and trust region policy optimization (TRPO). The entropy term (${\mathcal {H}}(.)$) is defined according to (29). The portion of entropy in the learning policy is determined by temperature parameter $\alpha$, which is decreased during the learning iteration as follows.

43 $$\begin{aligned}&\pi ^* ={argmax}_{\pi } \sum \limits _{t} \left({\mathbb {E}}_{(s_{t}, a_{t})}{\sim }_ {\rho \pi }\left[ r (s_t, a_t)+\alpha {\mathcal {H}}(\pi (a_t|s_t)\right)\right] , \end{aligned}$$

44 $$\begin{aligned}&{H}(\pi (a_{t}|s_{t})) = {\mathbb {E}}_{a{\sim }{\pi }(.\vert s)}\left[ -log(\pi (a|s))\right] . \end{aligned}$$

The entropy term will update the Bellman equation of the value network training process according to (45).

45 $$\begin{aligned} V(s_{t})= {\mathbb {E}}_{a{\sim }{\pi }_{\phi }(a_{t}|s_{t})}\left[ Q_{\theta }(s_{t},a)-\alpha log(\pi _{\phi }(a|s_t))\right] . \end{aligned}$$

The critic in SAC includes value function $V_\psi$ and soft Q-function $Q_\theta$. The actor contains policy network $Q_\phi$. The policy network in SAC chooses action from Gaussian probability distribution according to the squashing function $f_\phi$($\epsilon$;$s_{t}$) as follows.

46 $$\begin{aligned} a_t=f_\phi (\epsilon _t,s_t)=tanh(\mu _\phi (s_t)+\sigma _\phi (s_t)\epsilon _t),~~\epsilon \in {\mathcal {N}}(0,1). \end{aligned}$$

To use SAC for parameter estimation of the ADMM, we need to arrange the Markov decision process (MDP), including state, action, transition function, and reward. The state will be the decision variables of the ADMM problem, which are the Gasa island microgrid elements’ active and reactive power as follows.

47 $$\begin{aligned} S(t)=\{P^{g},Q^{g}, P^{l}, Q^{l}, u^{DG}, V_i, \theta _{i,j}\}. \end{aligned}$$

SAC will predict the suitable penalty parameter from the continuous action space according to (48), (49).

48 $$\begin{aligned}&100 \le \rho _{PQ,i} \le 10^4, ~~~~1 \le i \le n_{PQ}, \end{aligned}$$

49 $$\begin{aligned}&30 \le \rho _{V\theta ,j} \le 3000, ~~~~1 \le j \le n_{V\theta }. \end{aligned}$$

The reward function is defined as follow.

50 $$\begin{aligned} r(s_t, a_t)={\left\{ \begin{array}{ll} 200, &{}\quad if~ (37),(38),\\ 0, &{}\quad otherwise. \end{array}\right. } \end{aligned}$$

However, this reward function is a sparse reward. The NFP is a trick that is used in this study to empower stability and provision of efficient action space exploration to defeat the sparse reward issues^35^. A set of invertible functions establishes normalizing flows. With the change of probability distribution variables, normalizing flows sequentially transform a distribution to a more density distribution as follows^36^.

51 $$\begin{aligned} z_N = f_1 \circ f_2 \circ \cdots \circ f_{N-1} \circ f_N(z_0), \end{aligned}$$

where $z_{0}$ is the base distribution and $z_N$ is the final flow. The density of continuous variable the $z_N$ parametrized by $\phi$ is as follows.

52 $$\begin{aligned} ln p_{\phi }(z_N) = ln~p(z_{0})-\sum \limits _{n=1}^{N}ln~det|(\partial f_N/{\partial } z_{N-1})|, \end{aligned}$$

One of the simplest methods to determine invertible function f is RealNVP. We deployed the method introduced in^37^ to combine SAC and normalizing flows. We will reparametrize the Gaussian distribution of action selection with RealNVP invertible transformation as follows.

53 $$\begin{aligned} \pi (a|s)=tanh(f_1(\mu _\phi (s)+\sigma _\phi (s)\epsilon ) \circ f_N), \end{aligned}$$

where $\pi (a|s)$= $z_{0}$ and the log density of action is as follows.

54 $$\begin{aligned} log\pi (a|s)=log~p(z_0)-\sum \limits _{n=1}^{N}log~det|({\partial } f_N/{\partial } z_{N-1})|- \sum \limits _{n=1}^{N}log(1-tanh({(z_{N_n})}^2), \end{aligned}$$

**Figure 2:**
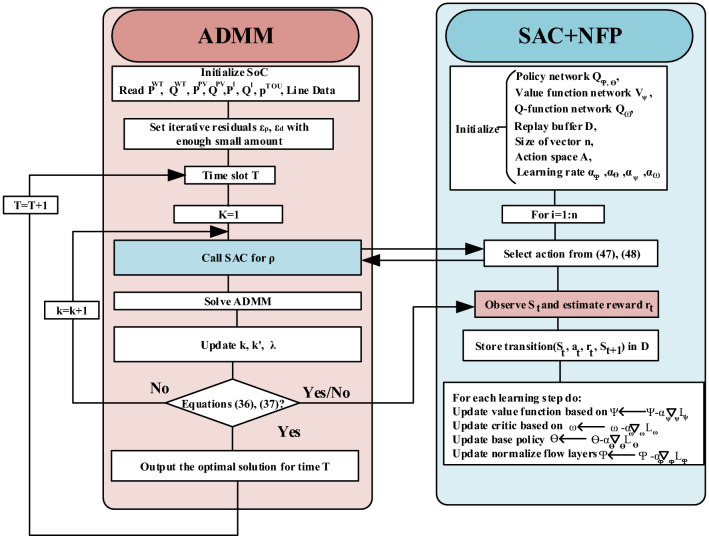
The proposed optimization algorithm for Gasa island EMS based on ADMM-NFP-SAC.

Therefore, the normal SAC will be modified by adding a gradient step on the normalized flow layers during the $\phi$ setting.

The proposed technique to determine penalty parameter of ADMM with the contribution of SAC and NFP is represented in Fig. 2.

**Table 2:** The Gasa island EMS solution simulation technical specifications and constraints.

**Microgrid elements**	
DGs	
$P_{DG,min/max}$ (kW/step)	75/330
$P_{DG,ramp-down/up}$ (kW)	120
$T_{DG,up/down}$ (h)	2
Quadratic coefficients	
$a_{1}$	1.3
(/kW)	0.0304
(/kW$^2$)	0.00104
RESs	
$P_{PV,max}$ (kW)	314
$P_{WT,max}$ (kW)	400
BESS	
$E_{bat}$ (MWh)	3
$P_{bat,min/max}$ (kW)	− 300/300
$SoC_{min/max}$ (%)	20/90
$\eta _{bat}$ (%)	90
$\sigma$ (KRW/MW)	100
$\eta ^{leakage}$	3%/month
Load	
$Temp^{in}_{min}$ ($^{\circ }$C)	23
$Temp^{in}_{max}$ ($^{\circ }$C)	25
Building thermal capacitance $\beta$ (kWh/$^{\circ }$C)	0.8
Building thermal reactance $\gamma$ ($^{\circ }$C/kW)	− 0.02
Heating and cooling anxiety rate coefficient (K)	100
Lower/Upper prefered time ($h_s$/$h_f$)	4/12 (a.m.)
Washing machine anxiety rate coefficients ($\sigma$, $\zeta$)	50
**Algorithm parameters**	
SAC	
Learning rate	0.0001
Discount factor ($\gamma$)	0.9
Replay buffer size	50,000
Number of training episodes	5,000
Mini-batch size	128
Number of hidden layer	2
$\alpha _{\phi }$, $\alpha _{\theta }$, $\alpha _{\psi }$, $\alpha _{\omega }$	0.0003
Activation function	ReLU
Optimizer	SGD
ADMM	
$\rho ^{PQ}_{min/max}$	100/$10^4$
$\rho ^{V\theta }_{min/max}$	30/3000
$\epsilon _{p}$, $\epsilon _{d}$	0.1, 0.001

**Figure 3:**
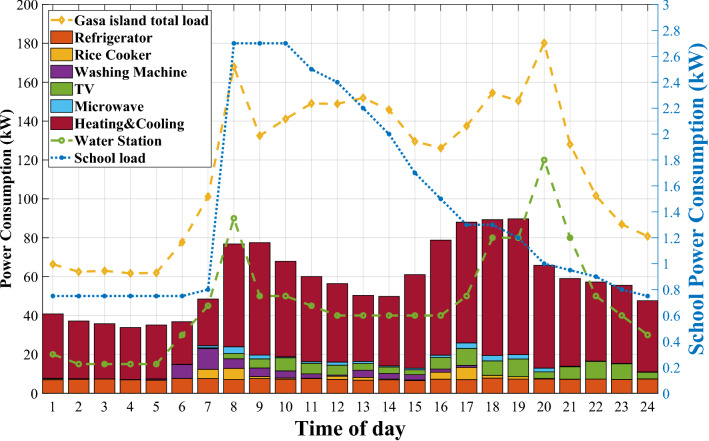
The Gasa island load profile based on consumers.

**Figure 4:**
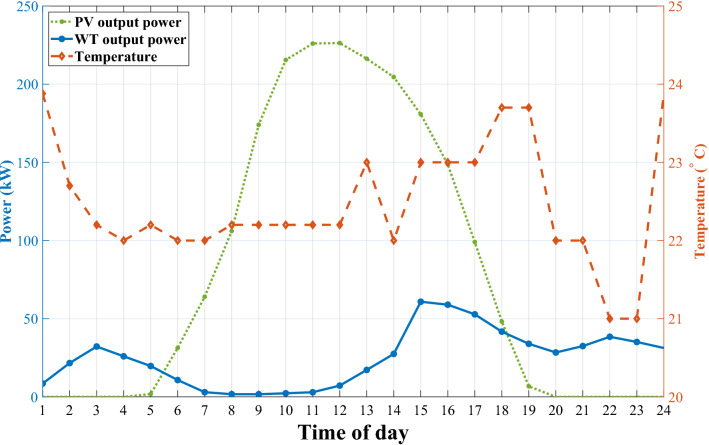
The Gasa island RESs output power and daily temperature during August.

**Figure 5:**
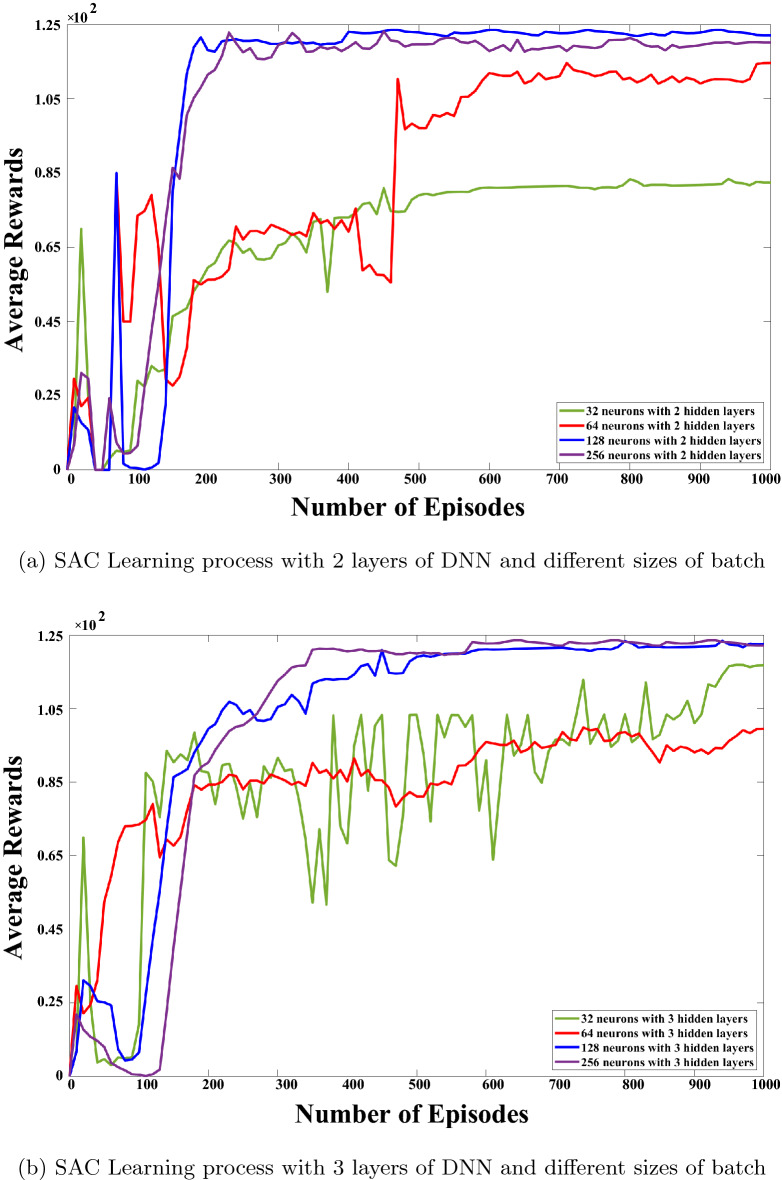
SAC hyperparameters justification.

**Figure 6:**
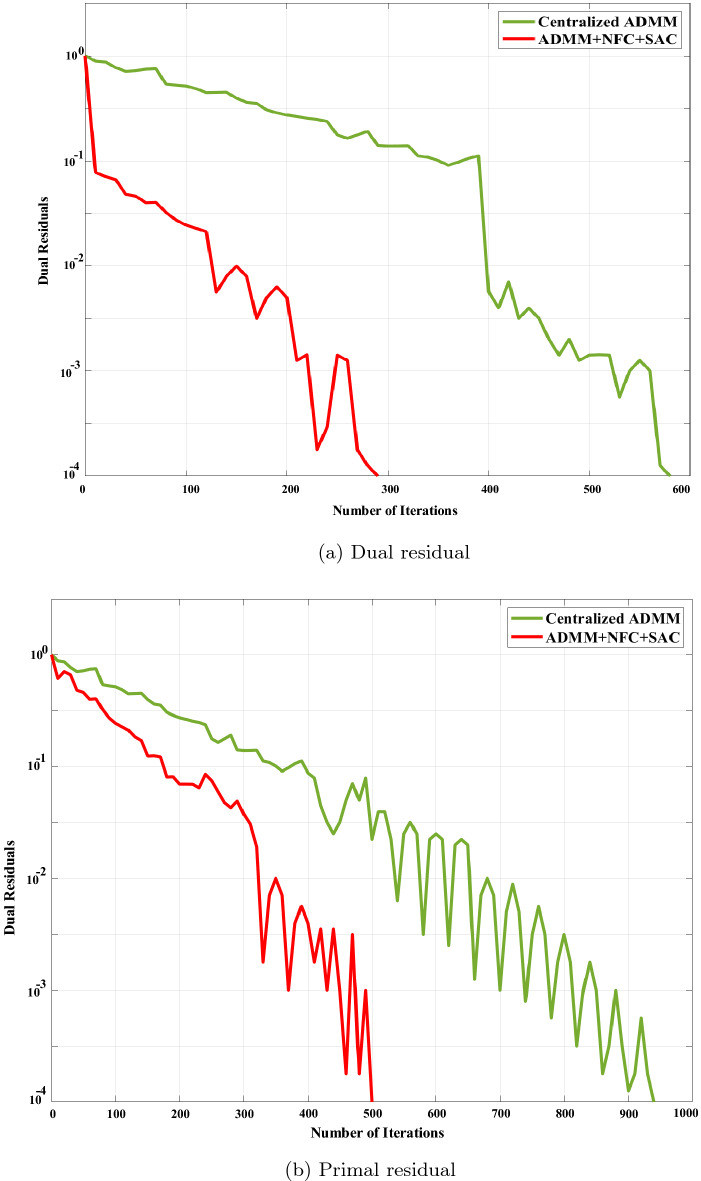
Convergence rate of residuals with centralized ADMM and proposed method comparison.

**Figure 7:**
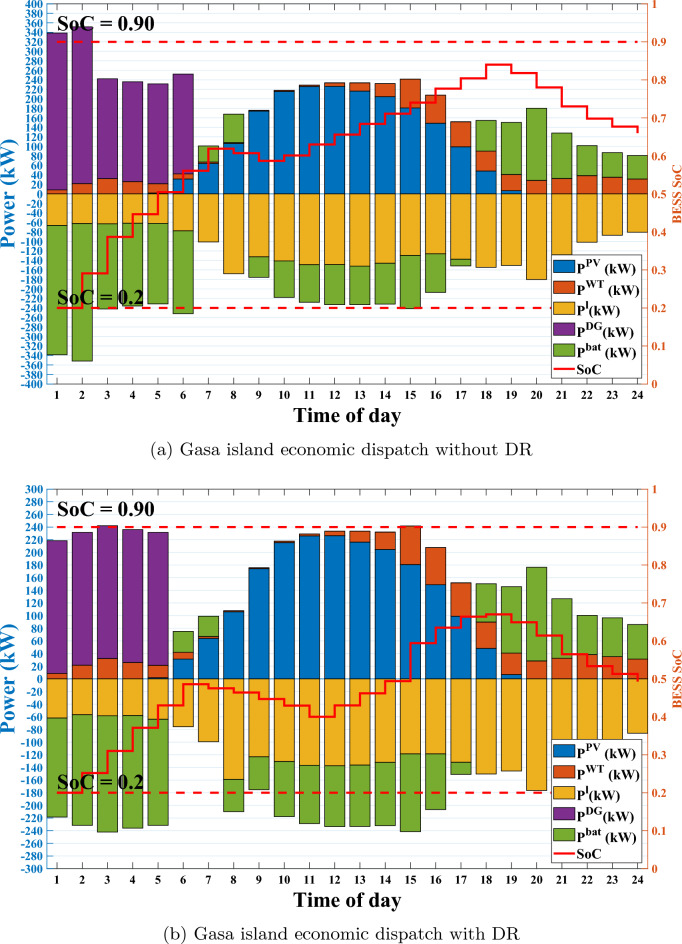
Economic dispatch in Gasa island microgrid with and without DR.

**Figure 8:**
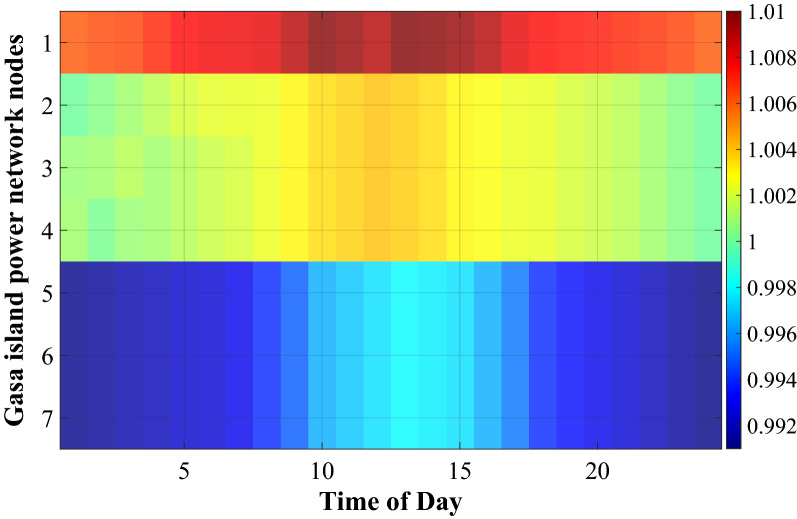
Gasa islands power grid nodes voltage magnitude (p.u.).

**Figure 9:**
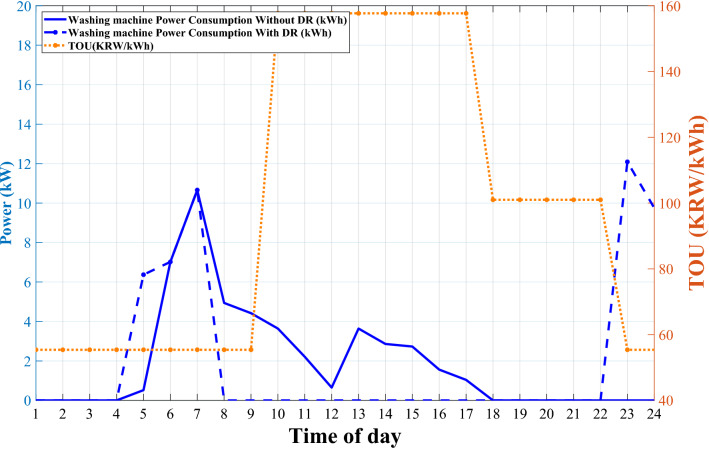
Washing machine power consumption with and without DR.

**Figure 10:**
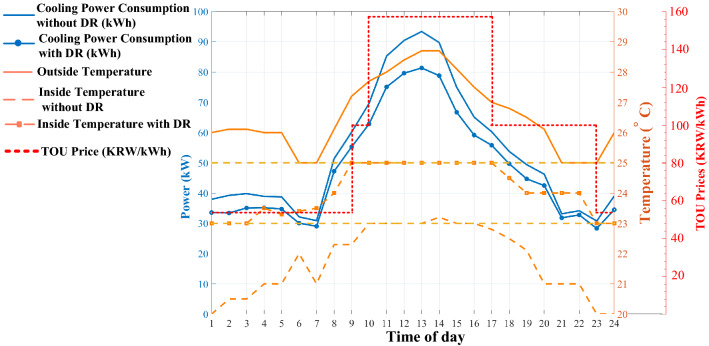
Cooling system power consumption with and without DR.

**Figure 11:**
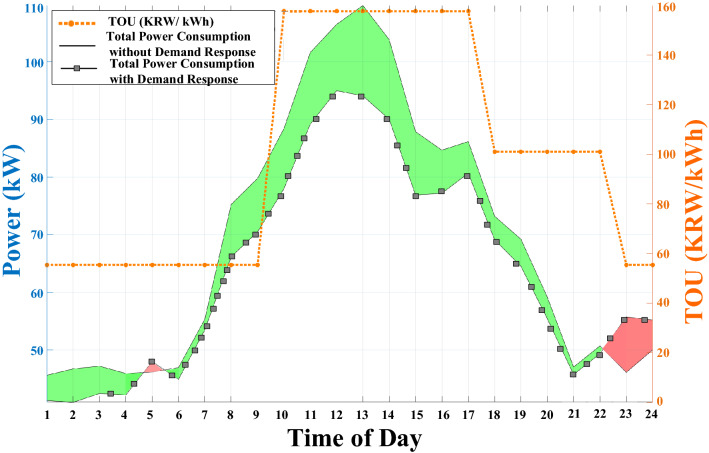
Total residential power consumption with and without DR.

**Figure 12:**
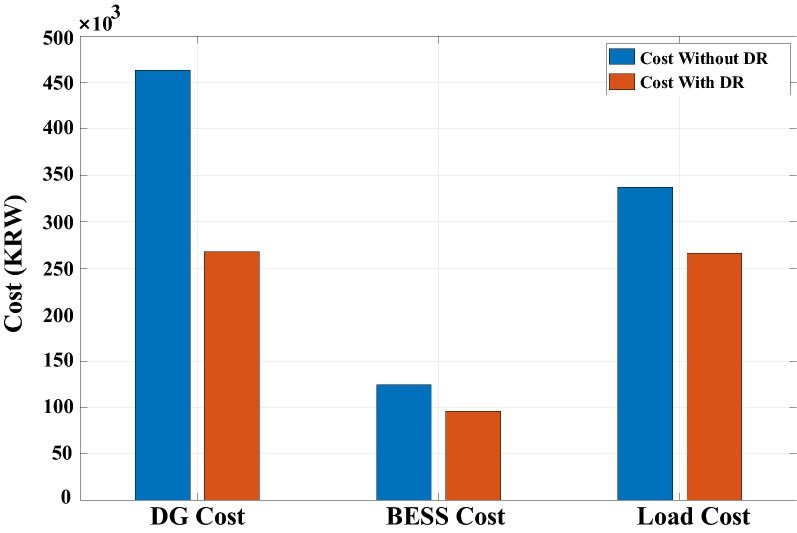
Comparison of DG, BESS, and load consumption cost for understudy day with and without DR.

**Table 3:** Comparison of proposed optimization method cost performance (KRW) with centralized-ADMM and CPLEX solver.

Optimization solution	Operational cost without DR (KRW)	Operational cost with DR (KRW)
CPLEX solver	832.88 $\times$ $10^3$	707.948 $\times$ $10^3$
ADMM	1091.441 $\times$ $10^3$	709.44 $\times$ $10^3$
SAC-NF-ADMM	924.95 $\times$ $10^3$	629.84 $\times$ $10^3$

## Results and discussion

We deploy our proposed algorithm to optimize the Gasa island microgrid EMS in this section. The optimization takes place hourly and can be extended to shorter intervals based on available data. Figure 3 depicts the load profile for the island power consumers based on “Microgrid elements modeling and constraints” section. Since the residential load profile is for August, we choose the WT and PV output power taken from^38^ and the output temperature^39^ according to this period, as shown in Fig. 4. We simulate the microgrid and implement a centralized ADMM solution as a baseline and our optimization solution with the CVXPY package^40^ to use OSQP solver^41^ in the Python environment. The simulations are accomplished on a PC with Intel(R) Core (TM) i5-10400F CPU @ 2.90GHz.

The SAC algorithm’s performance was compared with different hidden layer numbers and batch sizes to justify DNN parameters. Figure 5 shows how the learning process of DNN varies depending on hyperparameters. Based on the results, two layers with a batch size of 128 will result in a trade-off regarding learning convergence, stability, and complexity. It will not be advantageous to extend the network size to three layers and the batch size to 256 according to Fig. 5b. Table 2 depicts the microgrid element specifications and solution algorithm parameters.

Figure 6 demonstrates the convergence speed comparison of the proposed algorithm and conventional ADMM. The effectiveness of our hired technique appears in this figure, where surplus convergence of vanilla ADMM based on the number of iterations. By learning the best policy, the RL agent speeds up convergence for dual and primal residuals to 300 and 500 iterations, respectively, while for normal ADMM, this process time is double.

Figure 7 shows the Gasa island microgrid economic power dispatches along with the SoC level of BESS with and without DR. This figure illustrates the effectiveness of DR planning in reducing DG utilization during the understudy day. Without DR, the DG starts to work with a higher power generating amount of 315 kW at 1 a.m. to compensate for the shortage of RESs power shown in Fig. 7a. However, DR deployment causes this amount to reduce to 210 kW, as can be seen in Fig. 7b. Additionally, without DR, DG, due to ramp-down time limitations, should stay working on 315 kW, although the amount of shortage power is lower than this amount of generation. Moreover, we also solved the QP arrangement of Gasa island with the CPLEX solver as an analytical method. Table 3 represents our proposed SAC-NF-ADMM to improve the performance of ADMM in case of operational cost for under study day.

Figure 8 shows the voltage magnitude of the whole Gasa island power network nodes. This figure delineates the voltage magnitude of the island power network stay in the allowed range between 0.99 and 1.01 p.u.

The effects of DR scheduling on the cooling system and washing machine are represented in Figs. 9 and 10. The washing machine takes part in the DR program by adjusting operating hours to off-peak and mid-peak with lower TOU pricing. The results of DR implementation show that the anxiety rates perfectly direct the optimization problem to keep the desired time of washing machines’ working hours. As we discussed before, the washing machine’s preferred time of working is between 4 and 12 a.m. Therefore, as can be seen in Fig. 9, the washing machine power usage between 9 and 5 p.m. with higher TOU transferred to other times of day in the desired range.

The optimization algorithm justifies indoor temperature in the desired temperature between 23 and 25 $^{\circ }$C. The inside temperature tends to be higher between 10 a.m. and 5 p.m., where TOU is the highest amount, resulting in less power consumption for the cooling system compared to situations without DR schedules.

Figure 11 reveals the DR scheduling resulted in peak shaving of around 20% for residential load during peak hours. The green color shaded area in Fig. 11 shows peak shaving, while a red color shaded area signifies a transferred portion of peak load relating to washing machine usage. The most significant benefit of DR scheduling for consumers is dropping 21% in total consumer electricity bills, as illustrated in Fig. 12. After DR implementation, DG cost dropped by around 42%, which is another evidence of reduced DG usage and fewer gas emissions.

Here, we deployed the TOU policy to schedule DR scheduling currently utilized in the Korean power system. TOU is one of the price-based DR policies. It is also possible to implement DR on Gasa Island using other price-based DR models, such as real-time pricing (RTP) and critical peak pricing (CPP). Furthermore, incentive-based DR methods, including direct load control and emergency demand response programs (EDRP), can be used jointly with price-based DR with incentive payments from utilities to increase customer profits and encourage them to participate in DR. Therefore, in our future investigation, we will use a combination of price-based and incentive-based DR techniques to study their effect on $\hbox {CO}_{2}$ emission reduction. On the other hand, to completely meet the current situation of Gasa island, we deployed the WT and PV output power historical data. However, in our future attempt to consider uncertainties in WT and PV power generation, we deploy long short-term memory (LSTM) based solution to provide the RESs predictor.

## Conclusion

In this paper, we arranged the EMS unit for the Gasa island microgrid as one of the prominent Korean Green island project pilots. The proposed approach improves the main objective of this green island microgrid from the feasible framework for RESs utilization to a profitable microgrid for consumers with DR deployment. Additionally, our method resulted in less dependency on DGs with DR schedules. The ADMM-based solution for EMS provided a fast converged process of optimization by penalty parameter prediction with the state-of-the-art DRL method SAC. We released each iteration from the computational cost of transferring dual variable variants by definition of an independent, constant reward to each converged iteration. However, this approach resulted in a sparse reward hindrance for the process of training the agent. To overcome this problem, we used NFP to increase the probability distribution of policies. The results showed the proposed ADMM converged 50% faster than vanilla ADMM. Additionally, the implemented DR scheduling on reducible and shiftable residential load decreased 20% of the peak load. Since in this paper we considered current situation of Gasa island microgrid network TOU which is utilized DR policy in Korean power system conisdered to implement DR. In our future work, we will develop our study with utilizing CPP and EDRP. Furthermore, we will hire LSTM based predictor to estimate RESs output prediction.

## Data Availability

The datasets used and/or analyzed during the current study are available from the corresponding author on reasonable request.
